# Does Physical Activity Moderate the Relationship between Myopia and Functional Status in Children 9–11 Years of Age?

**DOI:** 10.3390/jcm11195672

**Published:** 2022-09-26

**Authors:** Monika Modrzejewska, Jarosław Domaradzki, Wojciech Jedziniak, Beata Florkiewicz, Teresa Zwierko

**Affiliations:** 1 II Department of Ophthalmology, Pomeranian Medical University, 70-111 Szczecin, Poland; 2 Unit of Biostructure, Faculty of Physical Education and Sport, Wroclaw University of Health and Sport Sciences, 51-612 Wroclaw, Poland; 3 Laboratory of Kinesiology, Functional and Structural Human Research Center, Institute of Physical Culture Sciences, University of Szczecin, 70-240 Szczecin, Poland

**Keywords:** myopia, balance control, schoolchildren, physical activity

## Abstract

Although previous studies have reported an association between physical activity (PA) and myopia in school-aged children, little is known about the link between myopia and children’s functional status. The purpose of this study was to investigate dynamic balance control in relation to the daily PA levels of myopic schoolchildren aged 9–11 years (*n* = 52) versus a non-myopic control group (*n* = 53). A single leg stance test performed on the instability platform of the Biodex Balance System was used to assess balance control. The overall stability index (OSI), anterior-posterior stability index (APSI) and medial-lateral stability index (MLSI) were analyzed. PA levels were calculated using the World Health Organization European Childhood Obesity Surveillance Initiative family record form. Myopes and non-myopes were separated into three subgroups based on PA level (low, moderate and high). Myopia significantly affected OSI (F = 40.46, *p* < 0.001), APSI (F = 33.93, *p* < 0.001) and MLSI (F = 49.51, *p* < 0.001). There were significant differences (*p* < 0.001) between myopic and non-myopic children with low and moderate levels of PA, whilst there were no differences between compared children with high levels of PA. High PA levels had a positive impact on balance control in myopes. Our results showed that PA levels moderate the relationship between myopia and children’s functional status.

## 1. Introduction

Myopia commonly develops in schoolchildren [1]. Previous studies have shown that myopia risk factors for schoolchildren include genetics [2], near visual tasks [3,4], time spent viewing screens [4,5], body stature [6], nutritional status [7], obesity [4,8], living environment and socioeconomic status [8], lower outdoor exposure [9,10], and race and ethnicity [11,12]. With the increase childhood myopia worldwide, myopia treatment and prevention has become a major public health issue, especially considering that high myopia (over –6.0 D) can cause pathological complications (i.e., cataracts, open angle glaucoma, retinal detachment, and myopic macular degeneration) [13].

Physical activity (PA) has a beneficial impact on physical and cognitive health in schoolchildren [14,15]. Childhood is a critical period for growth and motor development, and sedentary behavior during childhood is related to multiple health and development concerns that can lead to additional health consequences in adulthood [16,17,18], including problems related to myopia [19]. A number of studies have reported an association between PA and myopia in schoolchildren and adolescents and suggested that PA might be an independent environmental factor in relation to the development and progression of myopia [20,21,22,23,24]. It is currently believed that outdoor PA leads to a lower incidence of myopia and its progression among children by 13% to 50% [21,25,26,27]. Moreover, some studies have observed lower PA participation amongst children with myopia compared to non-myopic children [22,23].

To date, little is known about the potential link between myopia and motor skills. In one of the few examples of cross-sectional research on the subject, Lu et al. [28] found an association between myopia and aerobic capacity and muscular endurance in male military members. These findings suggested that the severity of myopia was negatively associated with participants’ physical fitness. To the best of our knowledge, the association between myopia and quantitative assessment of physical fitness in schoolchildren has not been examined. The current study was conducted to address this issue by investigating balance control in myopic schoolchildren. Balance is an important motor ability acquired during a child’s development and is a functional prerequisite for the performance of complex motor skills [29]. It is generally known that the visual system plays an essential role in balance control [30]. Moreover, several previous studies have indicated that experimentally induced myopia significantly worsens postural stability [31,32,33].

Based on gaps in the research, the aim of the present study was to investigate dynamic balance in relation to PA levels of myopic schoolchildren compared to a non-myopic control group of similar age and gender. Specifically, we aimed to: (1) assess the differences in balance task performance between myopic and non-myopic children, and (2) examine if PA levels moderate the relationship between myopia and the functional status of children based on interactions between PA levels and the presence or absence of myopia. In line with previous findings [16,18,23,28], we hypothesized that higher PA levels would be associated with lower deficits in functional status, and that myopia would negatively affect children’s functional status in a dynamic balance task [31,33]. We believe that understanding the potential effects of PA levels on the functional status of myopic children may help the development of strategies to prevent decrements in schoolchildren’s functional status.

## 2. Materials and Methods

### 2.1. Power Calculation

Before recruitment, a power calculation was conducted to determine the required sample size to detect medium-sized mediation effects [34]. Based on 85% total power, an α level of 0.05, and minimum effect size of 0.35, it was calculated that 105 participants would be required to detect a between-group difference in outcome values. To calculate the sample size, G*Power tool (Heinrich-Heine University, Düsseldorf, Germany) was used [35].

### 2.2. Participants

Eye screening tests comprised 1518 school-children aged 9–11 living in Szczecin, an urban region in Poland. In the group of 1518 children, post-cycloplegia myopia was confirmed in the group of 255 children, which constituted 16.8% of all subjects. Fifty-two (52) from the group of 255 children with myopia volunteered for the study, which indicates that the participation rate was 20.39%. Finally, 105 children aged 9–11 years (mean age = 10.14 ± 1.24 years) were recruited for this study. The participants consisted of 52 myopes (27 girls and 25 boys) and 53 non-myopes (33 girls and 20 boys). Amongst the myopes, 29 (55.77%) reported a family history of myopia. Mean visual acuity (assessed using a Snellen Chart and without correction) was 0.86 ± 0.24 for the right eye and 0.91 ± 0.21 for the left eye in the myopic group, and 1.0 ± 0.04 and 1.0 ± 0.03 for right and left eyes respectively in the non-myopic group (Table 1). All children underwent an ocular examination to assess objective refraction, binocular vision, stereoscopy (TNO test), and ocular motility including the presence of a latent and overt deviation for distant and near objects (cover test, Maddox test). Distance and near visual acuity was assessed using a Snellen Chart (with and without spectacle correction). Refraction was obtained using a hand-held Retinomax 3 autorefractometer (Righton, Tokyo, Japan) without and under cycloplegia with 1% tropicamide given three times every 10 min. The refractive error readings were expressed as the spherical equivalent (SE), which is the sum of the sphere power and half of the negative cylinder power and expressed in spherical diopters (SD). Refractions were categorized as myopia (≤−0.5 SD), emmetropia (>−0.5 SD to ≤+0.5 SD), mild hyperopia (>+0.5 SD to ≤+2.0 SD) and hyperopia (>+2.0 SD in children), astigmatism (≤−0.75 SD) and anisometropia (≥1.00 SD). Seven (13.46%) of the myopic children did not wear corrective eye-glasses daily. Table 1 presents the descriptive characteristics of the children.

### 2.3. Balance Tasks

The Biodex Balance System SD (Biodex Medical Systems Inc., Shirley, NY, USA) was used to evaluate balance control with eyes open. The system has 12 dynamic stability levels, with a highest stability level of 12 and a lowest stability level of 1. Balance control was measured during a dynamic task (i.e., single leg stance on preferred leg) with a platform instability level of 8. The total duration of the balance task was 80 s (three trials of 20 s with a rest interval of 10 s between each trial). For all trials children were tested barefoot. During testing, participants looked straight ahead with their arms folded across their chest. Myopic children were wearing glasses during the performance of the balance task. Before testing, three familiarity trials of 20 s with platform instability level of 8 was performed. The overall stability index (OSI, measured in degrees), the anterior-posterior stability index (APSI, measured in degrees), and the medial-lateral stability index (MLSI, measured in degrees) were analyzed. Higher stability index scores indicated poorer balance control.

### 2.4. Physical Activity (PA)

PA was measured using the Polish version of the World Health Organization (WHO) European Childhood Obesity Surveillance Initiative (COSI) family record form questionnaire. The family record form questionnaire was completed by participants’ parents or guardians, and as such was a proxy-respondent tool [36]. Information about a child’s PA consisted of: distance from school to home, transport from/to school (e.g., bus, car, foot, etc.), participation in sport clubs and extracurricular PA (hours per week), time spent on PA in child’s leisure time, time spent on homework, and sedentary behaviors (screen time).

For this study, a synthetic index of the PA (PASI) was calculated. It was constructed based on responses to each family record form questionnaire answer. All questions represented different components of PA and were accumulated into a common, agglomerated PA index. The agglomerated PASI was created using multidimensional comparative analysis (MCA) [37]. In order to ensure the comparability of the variables, a standardization process was carried out using the following formula:

$$z_{ij}=\frac{x_{ij}-{\bar{x}}_{j}}{s_{j}}$$

where *z_ij_* is the standardized value, *x_ij_* is the *j*-variable of the *i*-object, $\bar{x}$ is the mean value of the *j*-variable, and *s_j_* is the standard deviation of the *j*-variable [37].

After transformation, the diagnostic variables are standardized over the range [0; 1], which makes it possible to compare and estimate patterns and the distance from them [38,39].

MCA was used as each question measured a different dimension of PA, with answers recorded on different scales and with different units (e.g., days, hours, numbers, categorical). Thus, it was not possible to simply add results for each participant. The composite MCA index had to be constructed as a sum of components after converting each component according to the principles and rules of classification for multi-feature objects, as appropriate for MCA and linear ordering methods. These methods are popular in economics and econometrics, but have also been used in biological studies [37,38,39]. For different procedures, Hellwig’s pattern of development method was used. Detailed description of the pattern of development method was originally published by Hellwig [40].

Based on the PASI for each participant, terciles were calculated. The separate sets of terciles were used for the myopic and non-myopic groups. Initially, our concept assumed the same set of terciles for both groups (terciles calculated for non-myopes had to be implemented for myopes). The problem was that there were very unequal numbers of the participants in the subgroups of myopic children, with only three (3) persons with a high level of PA. This made it impossible to carry out a reliable analysis. Hence, we decided to apply two separate sets of terciles (calculated separately for both groups). Myopes and non-myopes were divided into three subgroups; low PA levels (below 1st tercile), moderate PA levels (between 1st and 2nd tercile), and high PA levels (above 2nd tercile).

### 2.5. Statistical Analysis

The Shapiro-Wilk test was used to evaluate the distribution of data, and all variables showed a normal distribution. Descriptive statistics for anthropometric measures and balance control variables are presented as means, standard deviations (SD) and 95% confidence intervals (CI).

Student’s *t*-test for independent variables was used to test for differences in morphological features between the myopic and non-myopic groups. Analysis of variance (ANOVA) was used to test for differences in PA levels between the three terciles in the myopic and non-myopic groups. Further comparisons were conducted using Tukey’s post-hoc tests.

For the main outcomes of OSI, APSI and MLSI, multivariate analysis of variance (MANOVA) was used to assess the significance of age and sex factors on the outcomes. Wilk’s *Λ* and *η*^2^ part (partial eta squared) were used to interpret results.

The significant impact of age led to the use of analysis of covariance (ANCOVA) for between-group comparisons. ANCOVA was performed twice. At the beginning a one-way ANCOVA with presence of myopia and as the main factor and age as a confounding variable (to hold the age effect constant) was used to compare the functional status of the myopic and non-myopic groups. Mean squares (MS), F statistics with accompanying *p*-values were calculated for each variable. Next two-way ANCOVA with presence of myopia and PA level as the main factors and age as a confounding variable was used to compare the functional status between six groups (myopic and non-myopic children with low, moderate and high level of the PA). Further comparisons between groups were conducted using Tukey’s post-hoc tests. Statistical significance was set at α = 0.05. Statistica version 13.0 (StatSoft Polska, Cracow, Poland) was used for data analysis.

## 3. Results

The small number of participants made it necessary to combine children regardless of age and gender within the myopic and non-myopic groups.

Comparison of the morphological differences between myopic and non-myopic children (separately for boys and girls) were conducted using independent t-Student tests. Compared to non-myopes, myopes were insignificantly (*p* = 0.58) higher, but there were significant differences in body mass (*p* < 0.001) and BMI (*p* < 0.001). Both groups of children were in the normal range for BMI (Table 1).

Non-myopes were more physically active (PASI mean = 0.475 ± 0.09) in comparison to myopes (PASI mean = 0.421 ± 0.07) and difference was statistically significant (*p* = 0.001). Based on individual results for the PASI, myopic children and non-myopic children were separated into three subgroups: low PA levels (below 1st tercile), moderate PA levels (between 1st and 2nd tercile), and high PA levels (above 2nd tercile). At the beginning the correctness of the division of groups into terciles was tested using ANOVA (this was to check that the terciles divided the groups appropriately). Significant differences between PA terciles (F = 119.07, *p* < 0.001) within each group (myopic and non-myopic) were observed, but differences between the same terciles across groups (F = 2.26, *p* = 0.135) were not found. Tukey’s post-hoc tests confirmed differences between subgroups. There were significant differences between low PA and moderate PA subgroups (*p* < 0.001), low PA and high PA subgroups (*p* < 0.001), and moderate PA and high PA subgroups (*p* < 0.001). However, significant differences in PA level were not observed between myopic and non-myopic children with low (*p* = 0.999), moderate (*p* = 0.999) and high (*p* = 0.112) PA levels. Although the ranges of the index are different (different terciles for both groups), the level of activity as measured by the PASI index within the subgroups is comparable.

The main outcomes were the balance control variables of OSI, APSI and MLSI. To decide which methodological strategy to adopt for between-group comparisons, a two-way multivariate analysis of variance (MANOVA) was conducted. The significance of sex and age on the outcomes was tested. A significant influence of age on the balance variables was observed (Wilk’s *Λ* = 0.874, *η*^2^ part = 0.065, *p* = 0.040), whilst there was no significant impact of sex (Wilk’s *Λ* = 0.994, *η*^2^ part = 0.006, *p* = 0.889). Taking into account the significant differences in BMI and age, ANCOVA with two confounding variables (controlling for BMI and age) was used to test the significance of the differences between subgroups.

The mean values and 95% CI for the balance variables in myopic and non-myopic children are presented in Table 2 (overall values). A one-way ANCOVA (one factor: presence of the myopia and age and BMI as a confounding variables) showed significant differences for all balance variables between myopic and non-myopic children (OSI: F = 28.24, *p* < 0.001; APSI: F = 27.00, *p* < 0.001; MLSI: F = 37.06, *p* < 0.001).

The next step in the analysis was to assess the moderating role of PA level on the balance variables. Descriptive statistics for OSI, APSI and MLSI in myopic and non-myopic children with different PA levels are shown in Table 2.

Results of the two-way ANCOVA (two factors: presence of myopia and PA level) adjusted for age and BMI are presented in Table 3.

Myopia significantly affected OSI (F = 40.46, *p* < 0.001), APSI (F = 33.93, *p* < 0.001), and MLSI (F = 49.51, *p* < 0.001), whilst PA level affected only OSI (F = 3.33, *p* = 0.040) and APSI, F = 4.95, *p* = 0.009). The impact of PA level on MLSI was not significant (F = 2.19, *p* = 0.118). The impact of myopia was higher than that of PA level in every balance test parameters. It confirmed higher values of MS (that can be treated as effect size parameter) for myopia factor than PA factor. The same parameter (MS) suggested the highest impact of myopia factor on OSI (MS = 24.73) compared to MLSI (MS = 19.57) and APSI (MS = 19.40). Significant interaction terms observed for the balance tests suggest however that the effect of myopia depended on the PA level, indicating that the impact of myopia varied across PA levels. Thus PA factor moderated the effect of myopia factor.

Further, detailed comparisons are presented in Table 4. Results of post-hoc tests confirmed a moderating effect of PA level on balance variables in comparisons between children with and without myopia with different PA levels. There were highly significant differences between myopic and non-myopic children with low and moderate levels of PA, whilst there were no significant differences between myopic and non-myopic children with high levels of PA. This applied to all balance variables.

## 4. Discussion

There are two important findings in this study: (1) the balance task results were poorer in myopic children compared to non-myopic children, and (2) the balance task results depended on PA level, so the impact of myopia changed across PA levels. Higher PA levels had a positive impact on dynamic balance task performance in children with myopia. Our results show that PA moderates the relationship between myopia and functional status in children aged 9–11 years.

The visual contribution to balance control strongly depends on the efficiency of the visual system. Previous studies have observed that experimentally induced myopia affects postural control. For instance, Paulus, Straube and Brandt [32] reported that when myopia was induced with spherical lenses of +4.00 D and +6.00 D, postural stability decreased by about 25% compared to control conditions. According to these authors, visual acuity, when decreased logarithmically, causes a linear increase in postural instability, and this is twice as prominent for fore-aft sway compared to lateral sway. Kim, Moon and Cho [33] concluded that even retinal blur caused by uncorrected myopia as small as −0.50 D can affect stability and balance. Moreover, in other studies, postural control in myopic groups (−3.00 D to −11.00 D) was assessed with and without corrective glasses [31]. It was observed that 25% more body sway occurred when participants did not wear corrective glasses. It is known that foveal vision, rather than peripheral vision, dominates postural control, particularly in swaying. That is why it is important to correct the refractive error and achieve full visual acuity (i.e., 1.0 on the Snellen Chart). Recently, it has been reported that postural stability was improved significantly when glasses were worn to fully correct each type of uncorrected refractive error (myopia and hyperopia) in young and elderly people [41,42]. It seems that correcting refractive errors might be one of the most basic strategies for improving balance control in children.

Next, it is also possible that the functional deficits in children with myopia may also contribute to oculomotor system disturbances. It has been reported that eye movement magnitudes are greater in highly myopic eyes compared to healthy eyes [43], and myopic patients tend to have poor fixation stability [44]. In our study, foveal fixation was present in all tested children, and ocular alignment measured by the Maddox test was within the normal range. However, a relationship between myopia severity and ocular misalignment may exist. It has been reported that exophoria is generally more common in myopic eyes than in non-myopic eyes [45]. Strabismus associated with myopia can be explained by one or several of the following mechanisms: anisometropia, alterations in the accommodation convergence/accommodation ratio (AC/A), and/or deviations in eye mobility muscle. Patients with progressive axial myopia (i.e., due to high axial length) exhibit increased accommodation amplitude in relation to an increased AC/A ratio. Fixation stability appears to be the strongest visual component affecting postural control [46,47]. In this context, further research examining eye movements and their interaction with the postural control system in myopic children is needed.

Our study results has shown that low PA levels had larger negative functional consequences in children with myopia compared to children without myopia. At the same time, higher PA levels had a positive impact on the functional status of children with myopia, and this status was comparable to children without myopia (Table 4). Deficits in balance control are an important intrinsic risk factor for falling and sustaining an injury in children [48], and compromise a child’s ability to master fundamental motor skills [29] and consequently their ability to participate in sporting activities. Balance control is a dynamic process developing across the life span, but it is usually poorer in children and seniors [48]. It was observed that static and dynamic postural control was significantly better in 19-year old healthy adults than in 7-year old children [49], mainly because a child’s neuromuscular system is not fully developed. Moreover, previous studies have shown that compared with well-sighted individuals, people with different kinds of visual impairment have lower levels of postural stability and thus have recommended PA interventions [50,51]. The protective role of PA and/or time outdoors against myopia in children and adolescents and the adverse role of a sedentary lifestyle have been reported in many studies [20,21,22,23,24,25,26,27]. Our study brings new knowledge to the field by showing a moderating effect of PA on functional status and gross motor skill performance in myopic children.

PA may have protective and stimulatory effects on the child’s functional status [14,15]. However, it has been indicated that PA and fundamental motor skills, such as balance, have a reciprocal and dynamic relationship, and a number of health-related factors and motor competencies might influence PA participation in childhood [52]. A number of studies postulate a cause-effect relationship between fundamental motor skills and PA [53,54], indicating that PA could stimulate functional development in childhood, which, in turn, results in an involvement in PA in the future. An area for further investigation is that of the relationship between the PA levels presented by children and the level of their functional status. In this work, we did not examine the above-mentioned relationship, but the results of our research indicate a potential link. The heterogeneity of the dynamic balance task results in the subgroups analyzed (presence of myopia and PA level) indicates the need for further analyzes that take into account the form of any relationships (e.g., multinomial rather than linear).

A fundamental limitation of cross-sectional studies is the inability to conclude any casual effect between analyzed factors. It is possible that the functional deficits of myopic children with low and moderate PA levels may have resulted from other factors that were not considered in this study. For example, preterm birth was not considered, yet it is assumed that children born preterm are more likely to develop myopia [55]. Moreover, in children born preterm various types of motor development deficits have been observed [56]. Thus, the possible visuomotor coordination deficits caused by preterm birth [57] may negatively impact balance control [58]. Secondly, we assessed children’s PA using a parents’ report questionnaire, which can lead to inflated estimates of activity. Using alternative tools for PA assessment (e.g., wearable accelerometer-based activity monitors) may increase the objectivity of PA measurement [59]. Thirdly, in our study, 13.46% (*n* = 7) of children did not wear corrective glasses or lenses daily, which may have a negative impact on their psychosocial well-being, classroom focus and participation, task persistence, academic achievements, and practicing school skills [60], including motor skills. However, we did not take this aspect into account in our analysis, and this should be addressed in future investigations.

In summary, this is the first study to demonstrate evidence of marked deficits in the functional status of myopic children aged 9–11 years. Taking into account good alignment of the eyeballs, no deviations in the form of overt and latent strabismus, and the presence of foveal fixation, it seems that for proper functional development refractive correction of any size of myopic error is essential. The results highlight that differences in dynamic balance task performance between children with myopia and children without myopia are moderated by their PA levels. Higher PA levels had a positive impact on dynamic balance task performance in children with myopia. These findings support PA programs for the maintenance or improvement of functional status in myopic schoolchildren. Future research should expand on these findings by exploring other potential explanatory variables that can have an impact on functional status in myopic children. In further studies, we propose an assessment of the relationship between PA levels and balance control in children with myopia compared to those without myopia.

## Figures and Tables

**Table 1:** Demographic and clinical characteristics of the children.

Parameters	Myopic Children		Non-Myopic Children	
	Mean	SD	Mean	SD
Age (years)	10.14	1.24	10.54	1.36
Height (cm)	139.94	6.39	139.30	5.48
Body mass (kg)	36.15	8.06	31.21	4.57
BMI	18.33	3.28	16.02	1.59
Distance visual acuity (RE)	0.86	0.24	1.00	0.04
Distance visual acuity (LE)	0.91	0.21	1.00	0.03
Visual acuity (near)	0.50	0.01	0.50	0.01
Spectacle correction (RE)	0.97	0.10	1.00	0.00
Spectacle correction (LE)	1.00	0.03	1.00	0.00
Spectacle correction (mean of both eyes)	0.98	0.51	1.00	0.00
Spherical equivalent (RE)	−2.08	1.38	0.17	0.11
Spherical equivalent (LE)	−2.14	1.74	0.23	0.05
Spherical equivalent (mean of both eyes)	−2.16	1.51	0.05	0.27
Spherical equivalent before the cycloplegia (RE)	−1.89	1.37	0.16	0.22
Spherical equivalent before the cycloplegia (LE)	−1.97	1.71	0.12	0.13
Spherical equivalent before the cycloplegia (mean of both eyes)	−1.93	1.44	0.14	0.17
Cylindrical equivalent before the cycloplegia (RE)	−0.48	0.45	−0.39	0.95
Cylindrical equivalent before the cycloplegia (LE)	−0.12	0.13	0.03	0.08
Cylindrical equivalent before the cycloplegia (mean of both eyes)	−0.46	0.31	−0.18	0.48
Spherical equivalent after the cycloplegia (RE)	−1.17	1.09	0.23	0.25
Spherical equivalent after the cycloplegia (LE)	−1.32	1.06	0.25	0.03
Spherical equivalent after the cycloplegia (mean of both eyes)	−1.25	1.01	0.24	0.13
Cylindrical equivalent after the cycloplegia (RE)	−0.54	0.99	0.37	0.13
Cylindrical equivalent after the cycloplegia (LE)	−0.41	0.24	0.00	0.00
Cylindrical equivalent after the cycloplegia (mean of both eyes)	−0.48	0.57	0.18	0.06
Maddox test	0.59	1.24	0.39	0.95

Note: RE—right eye, LE—left eye, BMI—body mass index.

**Table 2:** Descriptive statistics of OSI, APSI and MLSI in myopic and non-myopic children separated into PA levels.

PA Level	Stability Index	Myopic Group				Non-Myopic Group			
		Mean	−95%CI	+95%CI	SD	Mean	−95%CI	+95%CI	SD
Overall	OSI	2.11	1.82	2.40	1.04	0.98	0.84	1.13	0.54
	APSI	1.87	1.50	2.15	1.03	0.83	0.70	0.97	0.49
	MLSI	1.50	1.26	1.74	0.85	0.52	0.41	0.63	0.40
Low	OSI	2.43	2.02	2.85	0.95	0.86	0.40	1.31	0.71
	APSI	2.21	1.79	2.63	0.98	0.79	0.45	1.13	0.54
	MLSI	1.78	1.44	2.13	0.80	0.39	0.08	0.71	0.50
Moderate	OSI	2.18	1.62	2.74	1.12	1.00	0.75	1.25	0.49
	APSI	1.93	1.39	2.47	1.08	0.88	0.64	1.11	0.46
	MLSI	1.51	1.06	1.95	0.89	0.47	0.32	0.62	0.30
High	OSI	1.32	0.88	1.76	0.66	1.04	0.83	1.24	0.48
	APSI	1.05	0.67	1.42	0.56	0.83	0.61	1.04	0.51
	MLSI	0.90	0.50	1.30	0.59	0.62	0.45	0.79	0.41

Note: OSI—overall stability index, APSI—anterior-posterior stability index, MLSI—medial-lateral stability index.

**Table 3:** Results of the two-way ANCOVA adjusted for age and BMI (main effects: presence of myopia and PA level, interaction term effect).

Effect	OSI			APSI			MLSI		
	MS	F	*p*	MS	F	*p*	MS	F	*p*
Myopia (group)	24.73	40.46	<0.001	19.40	33.93	<0.001	19.57	49.51	<0.001
PA level	2.03	3.33	0.040	2.83	4.95	0.009	0.86	2.19	0.118
Myopia × PA level	3.40	5.56	0.005	2.91	5.09	0.008	2.48	6.27	0.003

Note: OSI—overall stability index, APSI—anterior-posterior stability index, MLSI—medial-lateral stability index, Myopia × PA level—interaction term.

**Table 4:** Tukey’s post-hoc results for balance scores between PA level in myopic and non-myopic children.

Variable	Pairwise Comparison								
	1 vs. 4	2 vs. 5	3 vs. 6	1 vs. 2	1 vs. 3	2 vs. 3	4 vs. 5	4 vs. 6	5 vs. 6
OSI	<0.001	<0.001	0.921	0.909	0.002	0.052	0.996	0.986	0.999
APSI	<0.001	0.001	0.966	0.836	0.001	0.034	0.999	0.999	0.999
MLSI	<0.001	<0.001	0.826	0.726	0.003	0.129	0.999	0.906	0.974

Note: 1—myopic low PA level, 2—myopic moderate PA level, 3—myopic high PA level, 4—non-myopic low PA level, 5—non-myopic moderate PA level, 6—non-myopic high PA level, OSI—overall stability index, APSI—anterior-posterior stability index, MLSI—medial-lateral stability index.

## Data Availability

Available upon request.
